# The Hybrid Nano-Biointerface between Proteins/Peptides and Two-Dimensional Nanomaterials

**DOI:** 10.3390/molecules28207064

**Published:** 2023-10-13

**Authors:** Giuseppe Forte, Diego La Mendola, Cristina Satriano

**Affiliations:** 1 Department of Drug and Health Sciences, University of Catania, Viale Andrea Doria, 6, 95125 Catania, Italy; giuseppe.forte@unict.it; 2 Department of Pharmacy, University of Pisa, Via Bonanno Pisano 6, 56126 Pisa, Italy; diego.lamendola@unipi.it; 3 NanoHybrid Biointerfaces Laboratory (NHBIL), Department of Chemical Sciences, University of Catania, Viale Andrea Doria, 6, 95125 Catania, Italy

**Keywords:** nanoparticles, adlayer, graphene, molybdenum disulfide, (M)Xenes, protein corona, 2D nano-biointerface

## Abstract

In typical protein–nanoparticle surface interactions, the biomolecule surface binding and consequent conformational changes are intermingled with each other and are pivotal to the multiple functional properties of the resulting hybrid bioengineered nanomaterial. In this review, we focus on the peculiar properties of the layer formed when biomolecules, especially proteins and peptides, face two-dimensional (2D) nanomaterials, to provide an overview of the state-of-the-art knowledge and the current challenges concerning the biomolecule coronas and, in general, the 2D nano-biointerface established when peptides and proteins interact with the nanosheet surface. Specifically, this review includes both experimental and simulation studies, including some recent machine learning results of a wide range of nanomaterial and peptide/protein systems.

## 1. Introduction

Nanomaterials offer unique chemical, physical, and mechanical properties due to their small size and large surface area. Hybrid biointerface-engineered nanoplatforms with proteins and peptides can pave the way for huge progress in several applications, such as nanomedicine, including, e.g., dual-targeted tumor hypoxia relief and enhanced photodynamic therapy [1,2], sensing [3], delivery, and release [4].

Graphene, since its discovery in 2004, has attracted great attention from the nanoscience field due to its remarkable optoelectronic, photothermal, physical, and chemical properties; it is the most exploited among the two-dimensional (2D) nanomaterials. Other 2D nanomaterials beyond graphene that have drawn much interest for bionanotechnology applications include graphene oxide (GO), reduced GO (rGO), and many other non-carbon-based nanosheets such as hexagonal boron nitride (h-BN), molybdenum disulfide (MoS_2_), and (M)Xenes.

While the studies on the electronic structures of 2D nanomaterials received huge attention in the last few years, the research on the interactions of 2D nanomaterials with biological molecules has lagged.

The urgency of research in this field arises from the fact that the hybrid nano-biointerface formed when biomolecules, especially proteins and peptides, adsorb onto nanomaterials, influencing their biological activity, stability, and overall performance. The existing gap of a deep understanding of the hybrid nano-biointerface established for 2D materials requests special attention, as biotic–abiotic interactions can be exploited to create novel multifunctional nanomaterials with more efficient electronic, optical, or catalytic properties.

As an example, biomolecule-tailored 2D nanomaterials, with the appropriate tuning of physicochemical features, are excellent candidates in many theranostic and nanomedicine applications, such as biosensors and drug delivery vehicles [5], and also in sustainable environmental recovery [6,7] and, in general, for nanotoxicant hazard management [8]. Furthermore, biogenic wastes (i.e., containing biomolecules) can play a role in the green synthesis of a different variety of 2D materials, which is of great relevance nowadays concerning the 2030 Agenda for Sustainable Development [9]. Moreover, the ‘decoration’ protein of nanosheets, such as GO and rGO, can offer new approaches, at suitable pH and temperature, for highly efficient assemblies of nanoparticles with a variety of compositions, sizes, shapes, and properties [10].

In physiological environments, as soon as an interface is established between a nanoparticle (NP) and a biological medium, the biomolecules that are present rapidly compete to bind to the NP surface, thus leading to the formation of a complex dress of biological molecules, referred to as the ‘corona’. The corona surrounding the NP surface largely defines the biological identity of the colloidal particle and is made up of the adlayer(s) of proteins, lipids, sugars, nucleic acids, and metabolites biomolecules, spontaneously adsorbed and bound either strongly or weakly onto nanomaterial surfaces and/or among each other [11]. To note, both the surface chemical structure, i.e., the chemical composition and spatial arrangement of atoms and molecules, and the topography, namely the micro-nanotextures and their characteristics, significantly influence the functional performance of any biointerface.

In the case of 2D nanomaterials, owing to the large specific surface area/mass ratio, referred to as specific surface area (SSA), and the high surface free energy, the adsorption of proteins and other biomacromolecules to form this corona is especially effective, affecting the interaction between the nanosheets and the biointerface when exposed to biological microenvironments.

Over the last five decades, the research on enhanced knowledge on the understanding of mechanisms of interaction between the biomolecule corona and the NP surface has been especially focused on the protein corona (PC), where the NP physicochemical properties, such as size and surface chemistry and the protein identity, have been identified as the main factors to drive the equilibrium binding between proteins and nanomaterial [12].

The biotransformation of the pristine nanomaterial surface on the spontaneous formation of PCs has a relevant impact on its pharmacological and toxicological profile, most often in an unpredictable manner. The PC covering nanomaterials in a physiological environment is the molecular interface that determines the response of the nanomaterial, mediating the interaction between the ‘bare’ NP surface and the biological system, thus influencing the mechanisms of action of the nanomaterial up to the sub-cellular level [13]. PCs affect the colloidal stability of the NP [14], the NP cellular uptake, and pathway activation [15]. PCs include both soft and hard coronas. The soft corona, containing proteins with low affinities to NP surfaces, undergoes reversible, dynamic exchanges, depending on the surrounding biological fluid. The hard corona is composed of proteins with substantial affinities to the NP surface and low tendencies to dissociate from it, which makes them relatively immobile in a shell layer that develops gradually [11]. PCs can inhibit endocytosis [16], which reduces the NP cytotoxicity [17] and influences the immunological response as well as the NP clearance processes [18].

In the context of the bioinspired and biomimetic approach, the harnessing of PCs around a 2D nanomaterial diminishes interaction with the plasma membrane by providing the nanomaterials with stealth properties [19]. This is especially relevant when the new ‘biological identity’ of the NP allows for thwarting interaction with some cell types (e.g., immune cells) and promotes efficient intracellular protein delivery [20,21]. Accordingly, several strategies have been outlined to harness PCs for driving the NPs’ biological fate and enhance the effectiveness of nanomaterials in nanomedicine, e.g., to meet the current challenges of poor pharmacokinetic properties, the off-target effect, and immunogenicity [13]. In general, tailoring the nanosheet surface with biomolecules has been exploited as a convenient strategy for different applications, including sensing and environmental management [7]. In this review, we overview some recent studies on the structures of proteins and peptides at 2D nanomaterial interfaces, including both experimental and simulation outputs of a wide range of nanomaterial and peptide/protein systems in the last decades.

## 2. The Nano-Biointerface between 2D Nanomaterials and the Biological Medium

**Scheme 1:**
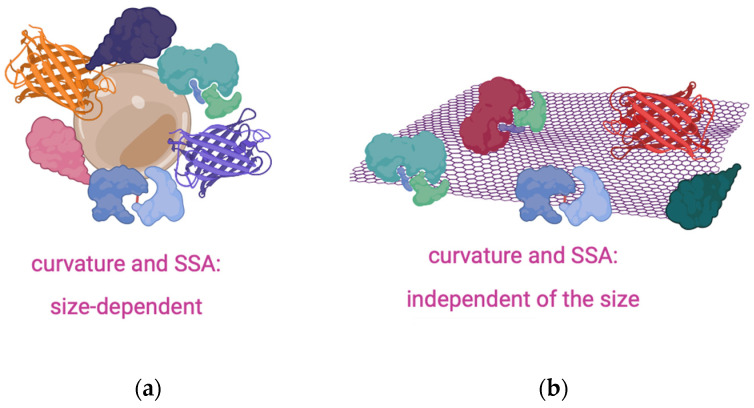
The protein corona dependance on the size of 3D (**a**) and 2D nanomaterials (**b**).

The 2D nanomaterials, i.e., those materials where the third dimension is almost negligible (approximately 1 nm per layer) than the other two dimensions, have a greater SSA compared to 3D nanomaterials. While the NP surface charge and chemistry unquestionably influence the PC formation and its spatial–temporal evolution, the size-dependence effect of curvature for 3D nanomaterials can be surely neglected for 2D nanomaterials, where the SSA is mass dependent but not size dependent [22] (Scheme 1). As an example, upon the exposure of GO nanoflakes of different lateral sizes (ranging from 100 to 750 nm) to human plasma, the PC formed around the nanoflakes did not depend on GO lateral size but was strongly influenced by protein concentration [23].

The physicochemical features are scrutinized for shedding light on the protein coronas onto the NP surface, and the most common experimental techniques used for this purpose include (a) the surface charge (by zeta potential, ZP); (b) the hydrodynamic size (by dynamic light scattering, DLS; fluorescence correlation spectroscopy, FCS; diffusion nuclear magnetic resonance, NMR); (c) the colloidal morphology and concentration (e.g., by absorbance spectroscopy; energy dispersive X-ray spectroscopy, EDS mapping; small-angle x-ray scattering, SAXS; electron microscopy, EM; atomic force microscopy, AFM); and (d) the PC composition (by gel electrophoresis, GE, proteomic mass spectrometry, MS; immunoblotting (e.g., western blots), and enzyme-linked immunosorbent assays, ELISAs) [24].

To assess the biological functions of the PCs, one can genrally measure (i) the bound protein structure and conformation (e.g., by circular dichroism, CD; solution NMR spectroscopy; sum frequency generation, SFG, vibrational spectroscopy); (ii) the protein–protein interactions and conformational changes (by fluorimetry and Förster resonance energy transfer, FRET); (iii) the protein stability and conformational changes (by nano differential scanning fluorimetry, nanoDSF); and (iv) the protein accessibility and function in the corona (by immunoblotting) [24].

Various computational methods, such as molecular dynamics (MDs), density functional theory (DFT), and coarse-grained simulations, have been employed to study protein/peptide–nanomaterial interactions. Recently, various effective in silico methods to predict PC composition on 2D nanomaterials by the use of machine learning (ML) algorithms have been developed [25].

### 2.1. NP’s Properties Affecting the 2D Nano-Biointerface

The size and surface properties of nanoparticles determine the protein corona with potential effects for biological influences [26]. In general, the composition of PCs strongly depends on the shape, size, and molecular composition of the NP, including the electrostatic charge, the hydrophobicity, the surface structure, the size, the curvature or shape, the stiffness, and, to a much lesser extent, the core material composition.

An elegant approach in recent developments has exploited and rationally designed the protein corona to achieve superior nanomaterial functionality, with a focus on preventing cytotoxicity and, in general, uncontrolled changes in the colloidal stability of the NP [24].

#### 2.1.1. Chemical Structure, Oxidation, and Wettability

Graphene and its derivatives can interact strongly with different serum proteins [27]. In particular, the GO, owing to its negatively charged oxygenated functional groups at physiological pH and hexagonal aromatic graphene structure, can interact with various proteins in the serum by promoting hydrogen bonding, electrostatic, hydrophobic van der Waals, and π–π (π stacking) interactions. A comparative study on the bovine serum albumin (BSA) corona and the human serum protein corona, formed on carbon black, multi-walled carbon nanotubes (MWNTs) and GO, pointed out that the serum PCs of all three different carbon-based nanomaterials are enriched with complement factors and apolipoproteins, with GO having the lowest affinity toward albumin and the highest absorptive capacity for other serum proteins [28].

The higher adsorption of serum proteins on GO than rGO are in the following order: bovine fibrinogen (Fg) > immunoglobulin (Ig) > transferrin (Tf) > BSA, which was attributed to differences in surface chemistry [29]. In particular, the GO’s polar groups such as hydroxyl, carboxyl, and epoxide promoted adsorption mainly through electrostatic interactions, whereas protein adsorption on rGO is mediated primarily by van der Waals interactions. Interestingly, a comparative study among GO and rGO, either partially reduced (i.e., having a low amount of oxygenated functional groups) or completely reduced (i.e., lacking oxygenated functional groups) hydrophobic graphene-like nanosheets, shows the highest adsorption of serum proteins on the partially reduced GO, which exhibits the optimal compromise of oxygen groups and hexagonal carbon structures [30].

The effect of the defect-induced hydrophilicity of MWNTs on the PC formation, as can be revealed using micro-Raman, photoluminescence, infrared spectroscopy, electrochemistry, and MD simulations, points out a strong influence of charge-transfer processes between the 2D nanomaterial and proteins (e.g., albumin and fibrinogen), which in turn induces protein unfolding and enhances conformational entropy, thus resulting in higher protein adsorption [31].

A study by proteomics analysis on the adsorption of plasma proteins onto Xenes, i.e., 2D monoelemental nanomaterials, including graphene, phosphorene, and borophene, showed that plasma proteins changed the surface identities of borophene nanosheets, with 46.5% of the 94 proteins bound being immune-relevant proteins [32]. In comparison with graphene and phosphorene nanosheets, 32 plasma proteins appeared on all three nanosheets. The proportion of immune-relevant proteins in graphene–corona and phosphorene–corona was 41.3% and 75.6%, respectively. The differences observed for the PC formed at the interface with the three Xenes can be explained considering the chemical nature of the elements constituting the nanosheets. Boron has a carbon-like sp^2^ hybrid orbital; therefore, for both borophene and graphene, stable low-dimensional nanosheets can be formed without being easily oxidized. On the other hand, phosphorene’s unique honeycombed fold structure allows for one phosphorus atom, the covalent bond, with three other monolayer P atoms, thereby exposing a lone pair of electrons, which are easy to react with oxygen to form oxides that are soluble in water.

A new family of 2D materials is MXenes, which are based on the carbide and nitride of transition metals with a general formula of M_*n*+1_X*_n_*T*_x_* (*n* = 1–3), where an early transition metal (M = Sc, Ti, Zr, Hf, etc.) is interleaved with n layers of carbon and/or nitrogen (X) with various surface functional groups (T = –OH, –O– or –F) [33]. While the hydrophilic nature of MXenes is widely reported, their surface architecture and chemical composition are determining factors in the ultimate wetting properties [34].

Three types of MXene Ti_3_C_2_T*_x_* nanosheets, prepared by different etching methods to grow diverse surface functional groups, were exposed to human plasma to scrutinize the protein corona by LC-MS/MS-based label-free proteomic analysis. The results revealed a significant difference in relative protein abundance for the three MXene nanosheets, with the main driving forces for the adsorption of plasma proteins on Ti_3_C_2_T*_x_* being identified as hydrogen bonding, steric hindrance, and hydrophobic interaction [35].

Using MD simulations, force field parameters were developed for MoS_2_ to reproduce the nanosheet experimentally measured water contact angle and predict the slip-length of water [36]. The results indicated that MoS_2_ consists of hydrophobic nanosheets and low-friction surfaces, despite the significant charges of surface atoms and relatively strong strength of van der Waals potentials [36].

#### 2.1.2. Surface Chemical Defects and Roughness

Proteins and peptides at the interfaces of nanostructured materials can be a result of their use in the synthesis and functionalization of nanostructures. Indeed, the chemical and structural properties of peptides and proteins allow their use as reducing, stabilizing, and functionalization agents [37].

Proteins and enzymes can be used as exfoliants by shear force to produce biofunctionalized nanosheet suspensions. In a study concerning the protein-induced layer-by-layer exfoliation process of various 2D nanomaterials, it was revealed by control experiments (TEM, AFM, XRD, UV-vis, Raman) and DFT simulations that benzene rings and disulfide groups of the BSA have much higher binding affinity to MoS_2_ nanosheets, while peptide bonds have much higher binding affinity to graphene compared to other groups [38].

During the functionalization and modification of graphene, chemical defects are often created, which typically alter the sp^2^ carbon hybridization, introduce defective sp^3^ carbon moieties, distort the hexagonal benzene ring structure of graphene, and affect protein binding due to steric hindrance and potential wrinkling of the graphene layer [39].

Several simulation studies on the role of graphene wrinkles and roughness revealed that rough graphene nanosheets trigger the adsorption of proteins, such as collagen fibers and peptides (RGD cell adhesive sequence), more than smooth surfaces due to the higher number of ‘contact points’ and reactive sites at the 2D nano-biointerface [40,41].

A protein-bonding layer functioned to dynamically tune the silver conductance in response to external pressures and strains and was prepared by the merging of silver nanoparticles under ambient conditions in an aqueous solution, with the formation of a freestanding large-area 2D silver film bound by ultrathin amyloid-like β-sheet stacking of lysozyme [42].

#### 2.1.3. NP Shape and Surface Curvature

Not only the chemical composition but also the shape of the NP is important in determining its interaction with proteins and peptides; indeed, the contacting surface curvature can lead to different adsorption capabilities. If the biomolecule’s size is much smaller than the substrate size, the surface curvature effects can be safely neglected. For NPs, whose size is comparable to proteins, the particle curvature must be accounted for by investigating the 2D nano-biointerface. Although the effect of the negative curvature of graphene surfaces is not fully understood, the protein adsorption onto the surface of carbon-based nanomaterials is known to be more prominent as the local curvature decreases. In fact, parallel fluorescence experiments and MD simulations for BSA adsorption onto carbon nanotubes of increasing radius (i.e., decreasing local curvature) and graphene (flat) nanosheets demonstrated an inverse relationship between protein adsorption capacity and surface curvature [43]. In a recent study, the adsorption of amyloid-β peptide (Aβ) onto graphene nanosheets with negative curvature was found by MD calculation to have a higher probability to adsorb than the one with positive curvature [44]. A dominant role in the adsorption process of Aβ onto graphene nanosheets has been identified in hydrophobic interactions and direct dispersion interactions [44].

A simulation study to account for nanostructured surfaces using the Random Sequential Adsorption model modified from protein adsorption on flat surfaces pointed out that convex geometries can lead to lower steric hindrance between particles and higher degrees of surface coverage per unit area [45]. A more pronounced effect was found in the case of a size mismatch between the proteins and the NP surface.

#### 2.1.4. Toxicity and Biofunctionalization

A study on GO nanosheets and the PCs formed by adsorption from fetal bovine serum (FBS), a commonly employed component in cell culture media, evidenced an FBS-mitigated GO cytotoxicity, arising from the hampering of direct interactions between the cell membrane and GO nanosheets that result in physical damage to the cell membrane [46]. Another recent study highlighted a convenient approach, based on laser-induced two-photon oxidation patterned GO surfaces, for controlled immobilization via non-covalent interactions of horseradish peroxidase and biotinylated BSA. AFM, Raman spectroscopy, and fluorescence microscopy confirmed the selective aggregation and the capability for localization of protein molecules onto the oxidized areas, as well as the tunability of the levels of oxidation [47].

Recent research findings evidenced that the adsorption of enzymes onto nanosheet surfaces of transition metal dichalcogenides, graphene, α-zirconium phosphate, and graphene oxide led to a decrease in the entropy of the enzyme’s denatured state and enhanced the stability of the bioengineered 2D nanomaterials [48]. Furthermore, the immobilization of cationized BSA protein to passivate the GO surface and other different types of nanosheets was exploited to make the nanosheet surface more biophilic, which allowed for increased loading, structure retention, and activity retention, e.g., in the case of the NP biofunctionalization with enzymes [49].

Studies on cytotoxicity, reactive oxygen species (ROS) production, and mitochondrial function disruption induced by rGO nanosheets coated with different concentrations of surfactants and ‘stealth’ functionalized with the protein corona formed in 10% FBS pointed out that the PC-coated rGO was more damaging than the bare nanosheets, inducing the most ROS and cellular death. The analysis of the nanomaterial and the PC correlated the more exposed rGO surface to higher lipid peroxidation, higher oxidative stress, and more cell death [50].

The adsorption of proteins on GO can also modify the nanosheets’ toxicity. GO functionalized with the angiogenin (ANG) protein represents a novel nanomedicine for modulating angiogenesis [51,52]. ANG is a powerful angiogenic factor [53,54] and a target for nanomaterial-based anticancer therapies [55,56]. The GO@ANG nanocomposite scrutinized utilizing UV-visible and fluorescence spectroscopies did not display cytotoxicity and was able to be internalized inside cells. In another example, for the application of GO as adsorption material in the removal of dye pollutants from water, the toxicity of GO has been demonstrated to be effectively attenuated using biopolymers, such as casein protein, to prepare GO biopolymer gels [57].

### 2.2. Advanced Research on the Hybrid Nano-Biointerface between Proteins/Peptides and 2D Nanomaterials

The 2D nano-biointerface is largely dependent on the biological environment, including biomolecular components, solution conditions, and surrounding temporal dynamics and hydrodynamics [58]. Binding affinities for the adsorption and desorption of proteins in the NP-PCs affect both the colloidal stability of the nanoparticle and the protein structures [14]. Proteins may change their conformational state upon adsorption onto NPs, depending on both protein and NP properties. In particular, structural changes can arise from interactions with the NP and also interactions between nearby proteins into the protein corona, leading to a dynamically evolving system as proteins continuously adsorb and desorb from the PCs. As an example, by properly directing the adsorption/desorption process, GO/SiO_2_ nanocomposites were successfully used as adsorbents for the selective adsorption/isolation of proteins of interest, namely hemoglobin (Hb), from complex sample matrices, such as the human whole blood [59].

Protein folding is a self-organized process that spontaneously occurs in the aqueous solution with high precision in a time length as short as milliseconds or fractions of seconds [60]. To note, the tertiary structure alteration of a specific protein within a PC is not a simple binary folded or unfolded process but is more nuanced; for instance, the folding of a protein may change forming β-sheets instead of α-helices [61].

It is not easy to understand this process considering the huge number of allowed conformations in the protein unfolded state, but there is agreement in identifying in the primary sequence the code of the folding [62]. However, the accumulation of partially folded conformations could hinder the folding process, resulting in the formation of macromolecular aggregates. Proteins can aggregate after they fold in the native state or via unfolded intermediate conformations. The aggregate formation may result in a very ordered process in which specific conformations are determined; the so-called amyloid fibrils display a β-sheet conformation, and their presence is a relevant hallmark in many pathologies such as amyotrophic lateral sclerosis, Alzheimer’s, and Parkinson’s diseases [63].

GO and graphene, with their atomic flat surface, the abundance of surface functional groups, and the ultra-large aromatic structure, are ideal platforms for elucidating, theoretically and experimentally, the immobilization mechanisms of biomolecules on them. It has been proved that enzymes, proteins, and short-chain peptides can be immobilized through either covalent or non-covalent interactions onto low-dimensional nanosheets [39,64,65].

The interaction of a protein or peptide with a surface can lead to modification of their native conformation and activity. On the other hand, more complicated supramolecular architectures can be assembled, paving the way for new applications. Peptides can form self-organized formations, such as fibers, rods, tubes, and ribbons, by tuning experimental conditions [66].

Greater changes in the protein/peptide conformation are observed in adlayers onto hydrophobic NP surfaces relative to their hydrophilic counterparts, with the shape of the protein impacting its stability on NPs with high surface curvatures. As the biological activity of a protein is strictly connected to its conformation and folded structure, even slight differences in the surrounding environment, such as pH and ionic strength, as well as the NP surface, may cause proteins either to lose or gain a function [61].

Significant structural rearrangement can be measured by CD spectroscopy analyses in BSA and Tf, from α-helical to enhanced β-sheet conformation, upon adsorption on GO nanosheets, while BSA unfolds on graphite surfaces, as expected on aromatic carbon surfaces [29]. The nano-biointerface of MoS_2_ nanosheet-protein complexes was studied using the four most abundant proteins in human blood, i.e., HSA, Tf, Fg, and immunoglobulin G IgG [18]. The quantitative analysis of protein coverage, binding affinity constants, and conformational changes in the PCs that IgG-coated and Fg-coated NPs triggered much stronger inflammatory effects of the MoS_2_ internalized by macrophages by producing and releasing more cytokines, although all of the MoS_2_-protein complexes induced inflammation [18]. The IgG, exhibiting the highest proportion of β-sheets, suffered minor secondary structure changes upon adsorption onto MoS_2_ nanosheets, triggering a stronger pro-inflammatory response in macrophages after the NP uptake due to the specific receptors recognition and the biochemical pathways activation [18].

In the case of the HSA-MoS_2_ nanosheet interface, as a consequence of ground-state complex formation by electron transfer, van der Waals, and hydrophobic forces, an altered conformation of HSA, as monitored by the quenching of the protein intrinsic fluorescence and the disruption of its binding domain with bilirubin, were found [67]. In addition, a decreased rate of the formation of the beta-sheet structures of HSA, a reduction in the non-enzymatic glycosylation activity, and an increased esterase-like activity of HSA were observed.

The interaction of the peptide with the interface and its orientation can induce a change in the conformational features of the free biomolecule. For example, the GAMHLPWHMGTL peptide sequence adsorbed onto graphene nanosheets by forming a complex reticular structure in a helical conformation that was different than the native α-helix structure [68].

Aromatic amino acids, such as phenylalanine (F) and tyrosine (Y), are known to assemble into nanotube or nanofiber structures via a process mostly driven by π–π stacking, charge transfer, and H-bonding [69,70,71]. FF micro- and nanotubes are known for their high mechanical strength, piezoelectric properties, and excellent functionalization capabilities, making them attractive materials for biosensing, tissue engineering, and energy harvesting applications [72,73]. An approach to stabilize FF nanostructures in water is to add GO that slightly affects nanotube morphology, maintaining self-assembly [74]. The comparison between FF and YY nanostructure formation onto GO highlights the effect of biointerface interaction [75]. The hydrophobic FF molecules preferentially gathered at the edges of the nanosheets, which contain a relatively low number of carbonyls, quinones, carboxylic acids, phenols, and lactones. Differently, the biphenolic-like YY molecules were found on the basal planes of the nanosheets, where GO is rich in phenol and epoxide groups, and the adsorption of Tyr is expected to be driven by electrostatic interactions and hydrogen bonding. The addition of a divalent cation, such as Cu^2+^, is able to form metal complexes with peptides and tune the formation of FF nanostructures, driving the aggregation on the GO sheets plane [75].

Dye-labeled peptide nanostructures assembled onto 2D nanomaterials have been used for the development of 2D bionanosensing devices. The dye-labeled peptide sequence HSSKLQ, sensitive to the prostate-specific antigen (PSA), can self-assemble onto GO via electrostatic and π–π interactions [76]. When the reaction with the proteolytically active PSA and the peptide adlayer are cleaved by the protease, recovery of the fluorescence originally quenched by electron transfer at the hybrid 2D nano-biointerface provides a quantitative readout for the proteolytically active PSA.

Noteworthy, the substrate-driven one-step aqueous self-assembly of lysozyme has been shown to be a cost-effective method to form a protein-based bilayer membrane that can efficiently recover precious metals from resources, including ores, waste electrical components, or wastewater [77]. To this respect, CD spectroscopy and scanning tunneling microscopy studies on the DELERRIRELEARIK sequence of a de novo designed α-helical peptide that can transform to β-sheet, as well as random coil on the addition of graphite particles to the peptide solution or aggregate into ordered β-sheet-rich assemblies at the graphite surface, are especially promising [78].

### 2.3. Basic Mechanisms and Peculiar Properties of the Nano-Biointerface: Driving Interaction Forces at the 2D Nano-Biointerface

In the complex interactions between 2D nanomaterials and proteins/peptides, the surface chemical structure, i.e., the atomic composition and arrangement of the outermost nanomaterial layer plays an important role in adsorption interactions, which may modify the secondary and tertiary structure of protein upon binding to the material surface [79]. In the interaction between biomolecules and nanomaterials, a variety of noncovalent forces come into play. These forces encompass hydrogen bonding, electrostatic interactions, hydrophobic interactions, π–π stacking, and van der Waals forces. Amino acids such as Ala, Phe, Ile, and Met predominantly partake in hydrophobic interactions, which intensify as the molecular weight increases. Conversely, charged amino acids such as Lys, Arg Glu, and Asp play a crucial role in van der Waals, electrostatic, and hydrogen bonding interactions and depend on the charge of the nanomaterial’s surface.

The π–π interactions predominantly occur between aromatic amino acids such as Phe, His, Tyr, Trp, and nanomaterials. These interactions arise from the delocalization of pi-electrons on the nanomaterial’s surface due to the presence of aromatic groups. The strength of these interactions is directly linked to the polarizability of the aromatic ring. Nanosheets such as GO feature a hydrophobic basal plane along with a hydrophilic edge and additionally exhibit a robust capacity for π–π interactions with aromatic amino acids, which significantly enhance protein binding [80,81,82].

In general, small peptides assemble preferentially to the edge or planar surface of GO or graphene via electrostatic or π–π interactions [83]. Based on theoretical studies, the strength of interaction between aromatic rings and GO surface has to be Trp > Tyr > Phe > His [84]. Experimental studies showed a different order, Arg > His > Lys > Trp > Tyr > Phe, highlighting the role of electrostatic interactions [85].

Studies carried out by paralleling SFG vibrational spectroscopy and MD simulations showed that the presence in a peptide sequence of planar sidechains containing charged hydrophilic (Lys), aromatic (Phe, Trp, Tyr, and His), amide (Asn and Gln), and guanidine (Arg) determines a preferential orientation of the peptide molecules adsorbed onto the graphene surface. The peptide interaction with graphene was demonstrated to be influenced by the competition between the peptide planar and hydrophilic residues [86].

A strong π–π interaction between GO’s basal plane and glucose oxidase has been demonstrated, along with the aromatic rings of amino acids aligning parallel to the substrate plane [87]. New quantum mechanical simulations have unveiled that proteins absorbed on the graphene surface change their secondary and tertiary configurations as a result of modification to the protein’s alpha helices. These interactions exhibit notable strength, causing aromatic amino acids to lie horizontally on the nanomaterial’s surface. This occurrence is responsible for the deformation observed in the protein’s helical arrangement and depends on the polarizability of the amino acid ring [88].

Schultz et al. have emphasized the significance of π–π stacking interactions between GO and biomolecules apigenin and orientin, as revealed through DFT calculations. Their investigation highlights that more stable arrangements arise when the structures are oriented parallel to each other. Electronic charge transfer confirmed that these weak interactions take place via a physisorption process [89].

In a recent study, Khedri et al. employed MD simulations to demonstrate the inhibitory impact of 2D nanomaterials against SARS-CoV-2. In all investigated scenarios, van der Waals interactions played a crucial role in the overall energy landscape. Notably, the spike protein was distorted by all categories of simulated 2D nanomaterials, leading to the reduced affinity for interacting with ACE2. Among these, functionalized (-) p-doped graphene exhibited the most effective performance, resulting in the inhibition of SARS-CoV-2 replication at the infection site [90].

**Figure 1:**
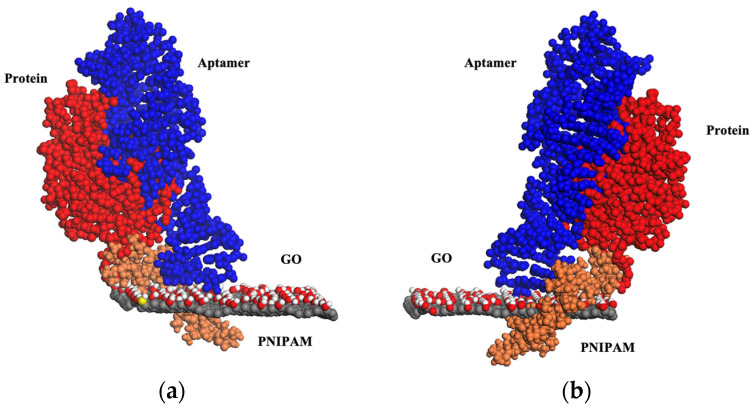
A schematic illustration, in two specular views, (**a**,**b**), of the interaction between the Wy5a aptamer and the α6β4 protein facilitated by PNIPAM attached to GO at 307 K. At this temperature, PNIPAM undergoes a transition into its globular form, fostering the aptamer–protein interaction.

Through molecular docking analysis, Unal et al. showed that GO sheets possess the capability to engage with surface components of SARS-CoV-2, thereby mitigating infectivity, even in the presence of any mutations on the viral spike. This study delved into the roles played by hydrogen bonds and hydrophobic interactions [91]. Using first principles and MD simulations, Putri et al. investigated a GO covalently grafted with PNIPAM [92,93,94] interacting with selective aptamers for cancer diagnostics. The findings revealed that the NIPAM monomer induces stability in the system through π–π stacking interactions between GO and the aptamer nucleobases. This establishment of stability facilitated a tunable surface between the Wy5a aptamer and the α6β4 protein without disrupting the central system’s electroconductivity. Furthermore, the thermo-responsive characteristics of PNIPAM led to an on/off surface state, thereby enhancing the interaction between aptamer and protein at a temperature above the PNIPAM LCST [95]; see Figure 1.

**Figure 2:**
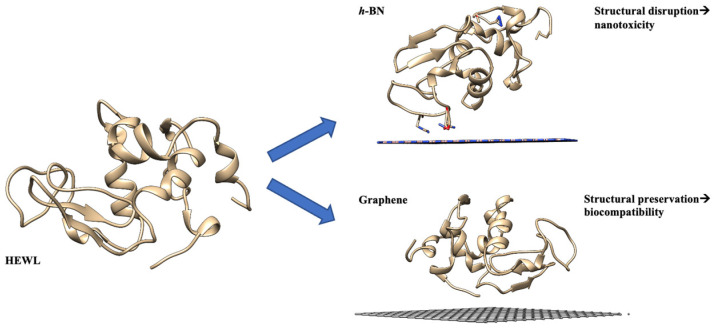
A schematic representation of the HEWL onto h-BN and graphene at the end of the simulation with different secondary structures.

Recent in silico investigations have been carried out to examine the nanotoxicity of 2D nanomaterials, like graphene and h-BN, toward the Hen Egg White Lysozyme (HEWL) protein. In this study, classical molecular dynamics simulations were employed. The results pointed to a notably stronger adsorption affinity of the protein onto h-BN in comparison to graphene. In this scenario, the protein’s native structure is significantly disrupted, whereas insignificant alterations are observed in the case of graphene. A strong perturbation of the secondary structure induced by h-BN can be attributed to van der Waals interactions and the electrostatic effect between the materials and aromatic amino acids. As a result of these interactions, the beta structure experiences complete diminishment. The study’s prediction indicates that h-BN might have noteworthy nanotoxicity toward proteins with a higher prevalence of aromatic amino acid residues. On the other hand, pristine graphene can be deemed a safer material when it comes to interactions with proteins [96]; see Figure 2.

Similarly, mutation studies by a combination of experimental methods and modeling and the bio-conjugation of BP7, a peptide sequence known with an affinity for h-BN, identified the Tyr residue as the key anchoring species, pointing to the importance of this residue in peptide/h-BN interactions. A further bio-conjugation of BP7 to a fatty acid allowed for the down-modulation of the Tyr contact to h-BN, resulting in the presentation of the extended peptide to the solution [97].

An MD study on the non-covalent interaction between the G protein and the hydrophobic surfaces of SWNT and graphene revealed that the G protein interacts with both surfaces, achieving a favorable dispersion in the media. Nonetheless, SWNT can influence the biological activity of the protein by reducing the alpha-helical structure, inducing an incorrect protein orientation and a diminished affinity toward its target. In contrast, graphene can appropriately orient the protein G toward its target, causing minimal structure alterations in the protein [98].

**Figure 3:**
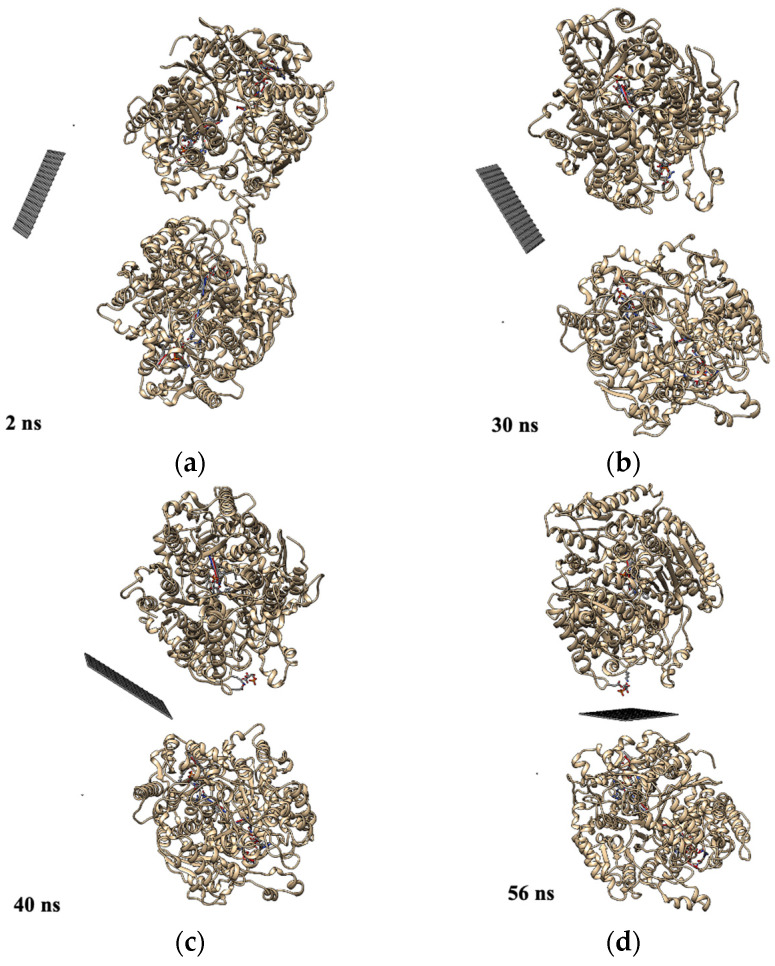
Dynamics of the insertion of a graphene sheet into the dimer at (**a**) 2 ns; (**b**) 30 ns; (**c**) 40 ns; (**d**) 56 ns (figure modified from Ref. [99]).

MD investigations have also demonstrated the graphene’s capability to elicit toxicity by interfering with protein–protein interactions (PPI). Simulations of PPI in the presence and absence of graphene showed that graphene inserted into PP led to the destabilization of hydrophobic interactions, disrupting the protein complex [99]; see Figure 3.

Molecular dynamics simulations were carried out to study the interface between neurotrophin-mimicking peptides [100,101,102] non-covalently linked to the surface of GO [103]. Among the peptides under investigation, NGF (1–14) exhibited the strongest interaction with the GO substrate. This strong interaction was primarily attributed to hydrogen bonding between the hydrophilic surface of the arginine and glutamic acid residues.

**Figure 4:**
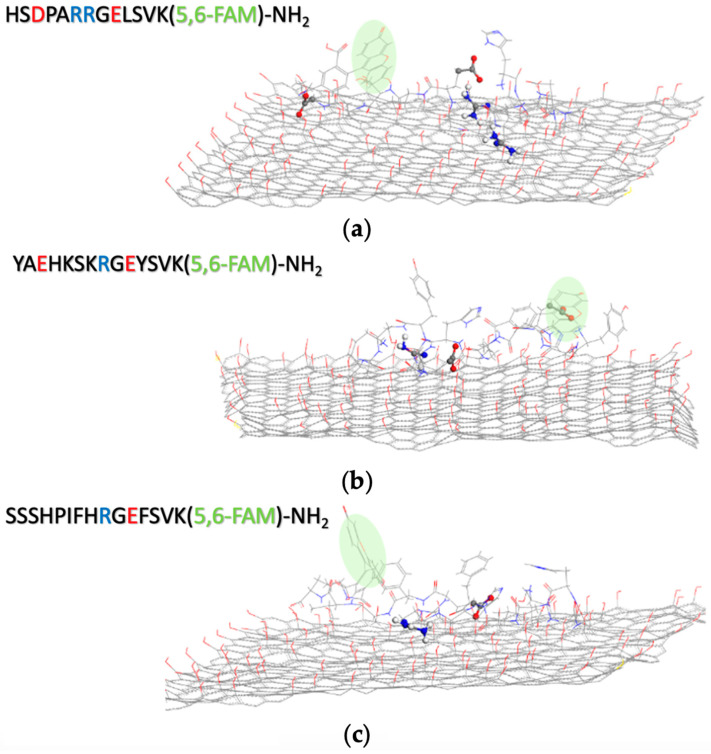
Simulation models for the interaction between GO and (**a**) BDNF (1–12), (**b**) NT3 (1–13), and (**c**) NGF (1–14). Water molecules are omitted for clarity, and carboxyfluorescein moiety is highlighted in green (from Ref. [103]).

Notably, the glutamic acid residue assumes a crucial role since this moiety appears highly distanced from the surface in the case of BDNF (1–12), which coincides with the peptide displaying the lowest interaction energy; see Figure 4.

Being a trailblazing non-graphene substance, the single-layer 2D MoS_2_ has demonstrated remarkable achievements in the realm of biosensor research.

**Figure 5:**
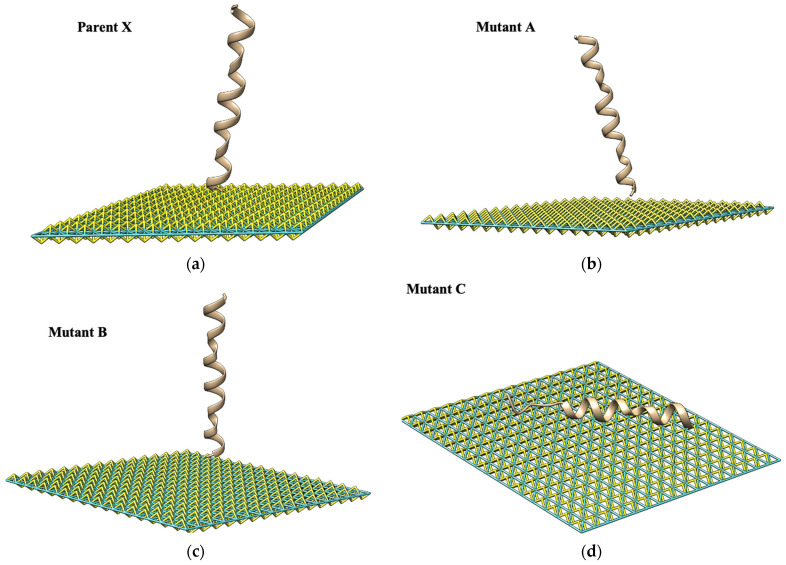
A schematic representation of the predicted structures for the peptide adlayers. In panels (**a**–**c**), the tilting of the parent peptide X (KWKLFKKIGIGAVLKVLTTGLPALIS), mutant A (KAKLAKKIGIGAVLKVLTTGLPALIS), and mutant B (SWSLFSSIGIGAVLKVLTTGLPALIS) are displayed. In panel (**d**), the parallel configuration of mutant C (KWKFFKKIGIGAVLKVLTTGLPALIS) on MoS_2_ is shown, with the helicity of its secondary structure conserved during the simulations. Water is not shown for clarity.

Song et al. employed first-principle calculations to scrutinize the adsorption mechanisms of aromatic amino acids (Phe, Tyr, and Trp) onto monolayers of pristine MoS_2_ or Au-modified MoS_2_ surfaces. In the case of pristine MoS_2_, the calculations unveiled adsorption via parallel interactions between the aromatic rings of the biomolecules and the surface sulfur atoms. For the Au-modified MoS_2_, a combination of chemi- and physisorption was found, with covalent attachment or non-covalent interactions toward Au atoms or the MoS_2_ sheet surface, respectively. In both cases, the adsorption energy followed the order Trp > Tyr > Phe [104]. A new force field based on quantum chemical data, called MoSu-CHARMM, has been developed to describe the non-covalent interactions between the MoS_2_ surface and a wide range of chemical entities encompassing amino acid groups. Data obtained using this force field are in excellent agreement with the experimental structural data of peptides adsorbed at the aqueous MoS_2_ surface, reproducing the helical secondary structure in the surface-absorbed state [105]; see Figure 5.

Furthermore, all atom MD simulations of the interaction between a MoS_2_ nanosheet and a model protein used in protein folding studies, the Villin Headpiece (HP35), pointed out that for the protein anchoring onto the MoS_2_ surface, aromatic and basic residues play a pivotal role, which, in turn, prompt the following protein unfolding [106]. The MoS_2_ nanosheet exhibits a denaturing capability toward HP35 (within hundreds of nanosecond simulations), and the main driving force behind the adsorption was identified in the dispersion interaction between the protein and the nanosheet. Furthermore, a synergic effect from H_2_O molecules at the interface between some key hydrophobic residues (e.g., Trp-64) and MoS_2_ surface triggered the secondary structure disruption by providing a strong hydrophobic force driven by nanoscale drying.

Differently from graphene and GO surfaces, the aromatic amino acids do not have a substantial effect on peptides interacting with the MoS_2_ surface [107]. The systematic study using the peptide sequence KWKLFKKIGIGAVLKVLTTGLPALIS and different single-point mutated analogs showed that the charged groups in the N-terminus region were essential to determine peptide interaction on MoS_2_. The facet edges of MoS_2_ were utilized as a landmark to identify the crystallographic orientation of the self-assembled structures of l/d-type peptides on molybdenum disulfide [108]. Both peptide enantiomers formed nanowires on MoS_2_ with mirror symmetry, according to the facet edges of MoS_2._

A selection of the recent literature on 2D peptide nanomaterial assemblies for fabricating functional nanoplatforms for various applications is provided in Table 1.

## 3. Conclusions

The ultrathin structure of nanosheets, the high aspect ratio, surface area, and surface free energy, enable them to more easily penetrate biological membranes and promote the adsorption of drugs and proteins, which originates the peculiar response at the biointerface and many potentialities for the application of 2D nanomaterials in the biomedical field. However, even though in vitro studies are very promising, one of the main reasons for the wide gap between benchtop discoveries and clinical practice, other than toxicity and scale-up issues, is certainly our limited knowledge about the physicochemical transformation of the 2D nanomaterial in vivo. The investigation and thorough understanding of the structure−performance relationship of the hybrid 2D nano-biointerface can pave the way for novel material design aiming at special functions and applications, not only in nanomedicine but also in environmental management, such as eco-friendly systems to recover precious metals from resources and pollutant removal from aqueous environments.

This report covers the basic information and the most emerging applications of the nano-biointerface established between 2D nanomaterials and proteins/peptides. In particular, regarding both experimental and computational points of view, we focused on the structure–property correlation of the bioengineered nanocomposite material formed when biomolecules are adsorbed/grafted onto the nanoparticle surface. Recommendations for future studies include the emerging areas of bioinspired chemistry on multifunctional bioengineered 2D nanoplatforms for multimodal theranostics and smart, stimuli-responsive systems for sustainable, eco-friendly approaches to environmental applications regarding contaminant removal and recyclability.

## Figures and Tables

**Table 1:** Some recent studies on 2D nano-biointerfaces established between peptides and nanosheets.

Nanomaterial	Peptide Core Sequence and/or Name	Lead Interaction	2D Platform Application	Ref.
GO, GQD ^1^	395-KKG; 395-G	covalent	Immunosensing for leishmaniasis detection	[109]
GO	FEFKFEFK	electrostatic	3D injectable cell delivery	[110]
GO	GRPR ^2^ antagonists bombesin (BBN) peptides: GGGRNFQWAVGHL (MATBBN); FQWAVGHL (ATBBN)	electrostatic; π stacking	pH-sensitive drug release	[111]
GO, CNTs ^3^	Boc-FF; Boc-YY	π stacking	Photocontrolled drug release	[112]
GO	YSAYPDSVPMMS (YSA); SWLAYPGAVSYR (SWL)	covalent	Targeted therapeutic drug for non-small cell lung cancer	[113]
graphene	Silk amino acids	H-bonding; electrostatic	Composite scaffold for peripheral nerve regeneration	[114]
GO	KKNYSSSISSIHC (dye-labeled LPS-binding peptide) ^4^	electrostatic	Fluorescent turn-on sensor for endotoxin detection	[115]
GO, rGO	VEVKVEVK (V8); FEFKFEFK (F8); FEFEFKFE (FE)	π stacking	Hybrid peptide hydrogels for biomedical applications	[116]
GO	RRRRNLWAAQRYGRELRRMSDKFVD (R4); RRRRRRNLWAAQRYGRELRRMSDKFVD (R6); RRRRRRRRNLWAAQRYGRELRRMSDKFVD (R8)	electrostatic; π stacking	Fluorescence ‘turn-on’ protein sensing	[117]
GO	FLGVVFKLASKVFPAVFGKV (D28)	covalent grafting	AMP ^5^-based nanomaterial against *Candida albicans* and *Escherichia coli*	[118]
GO	ε-poly-L-lysine	electrostatic	Removal of waterborne contaminants; superbug eradication	[119]
GO	CGGHSSKLQFWYFWY	electrostatic	Electrochemical biosensor for the sensitive analysis of PSA ^6^	[120]
GO	SWVGKHGKKFGLKKHKKH (AWRK6)	electrostatic; π stacking	AMP-based nanomaterial for the neutralization of endotoxin activity in the treatment of sepsis	[121]
GO	YIGSRWYQNMIRIKVAV (PepS1); QHREDGSYIGSRIKVAV (PepS2); WQPPRARIYIGSRIKVAV (PepS3); DGEARGDSPKRSR (PepS4)	electrostatic; π stacking	Multifunctional osteogenic differentiation of WJ-MSCs ^7^ in bone tissue regeneration	[122]
rGO	EPLQLKM- S protein (SARS-CoV-2-antibody binding site)	electrostatic; π stacking	Electrochemical immunosensor for the SARS-CoV-2 virus	[123]
MoS_2_	EYGAGAGAGAGAYE (EY5); RYGAGAGAGAGAYR (RY5); QYGAGAGAGAGAYQ (QY5); YGAGAGAGAGAY (Bio-Y5Y)	electrostatic	Biomolecular scaffold for biosensing	[124]
MoS_2_	GIGAVLKVLTTGLPALISWIKRKRQQ (melittin)	covalent grafting	An AMP ^5^-based theranostic nanoplatform for PTT ^8^ and PDT ^9^ processes in superbug inactivation	[125]

^1^ GQD: Graphene Quantum Dot. ^2^ GRPR: gastrin-releasing peptide receptor. ^3^ CNTs: oxidized carbon nanotubes. ^4^ LPS: lipopolysaccharide. ^5^ AMP: antimicrobial peptide. ^6^ PSA: prostate-specific antigen. ^7^ WJ-MSC: Wharton’s jelly-derived mesenchymal stem cells. ^8^ PTT: photothermal therapy. ^9^ PDT: photodynamic therapy.
